# Correction to “Obstructive Sleep Apnea in Overweight and Obese Children: Factors Influencing Quality of Life”

**DOI:** 10.1002/lio2.70179

**Published:** 2025-06-22

M. Al‐Iede, R. Rahal, S. Al‐Mashaqbeh, et al., “Obstructive sleep apnea in overweight and obese children: factors influencing quality of life,” *Laryngoscope Investig Otolaryngol* 10, no. 3 (2025), e70134. doi: https://doi.org/10.1002/lio2.70134.

The affiliation of the last author, Nihad Almasri, is incorrect and should be replaced with: Department of Rehabilitation Sciences, College of Health Sciences, QU Health, Qatar University, Doha, Qatar.

The second email address of the corresponding author, Montaha Al‐lede, is incorrect. It should be replaced with m.al-iede@ju.edu.jo.

We apologize for these errors.

